# A silver NP-dispersed water extract of fly ash as a green and efficient medium for oxidant-free dehydrogenation of benzyl alcohols†

**DOI:** 10.1039/c7ra12225j

**Published:** 2018-01-02

**Authors:** Bishal Bhuyan, Arijita Paul, Meghali Devi, Siddhartha Sankar Dhar

**Affiliations:** Department of Chemistry, National Institute of Technology, Silchar Silchar-788010 Assam India ssd_iitg@hotmail.com +91-3842-224797 +91-3842-242915

## Abstract

Herein, a green, efficient, and new catalytic system for dehydrogenative oxidation of benzyl alcohols using Ag nanoparticles (NPs) dispersed in water extract of fly ash (WEFA) has been developed. Various characterization techniques were performed to authenticate the formation of Ag@WEFA. The as-prepared Ag NPs (10–20 nm) were found to be dispersed in WEFA, as indicated by transmission electron microscopy (TEM) and scanning electron microscopy (SEM) images. With Ag@WEFA, a variety of substituted benzyl alcohols were efficiently converted to carbonyl compounds in high yields. All the reactions were deliberately carried out without using any ligand or hazardous organic solvent. This catalytic system involving WEFA is a genuinely new concept. It is, therefore, expected to attract attention from researchers working in the areas of sustainable chemistry.

## Abstract 2

A new catalytic system for dehydrogenative oxidation of benzyl alcohols using Ag nanoparticles dispersed in WEFA is developed.

## Graphical abstract

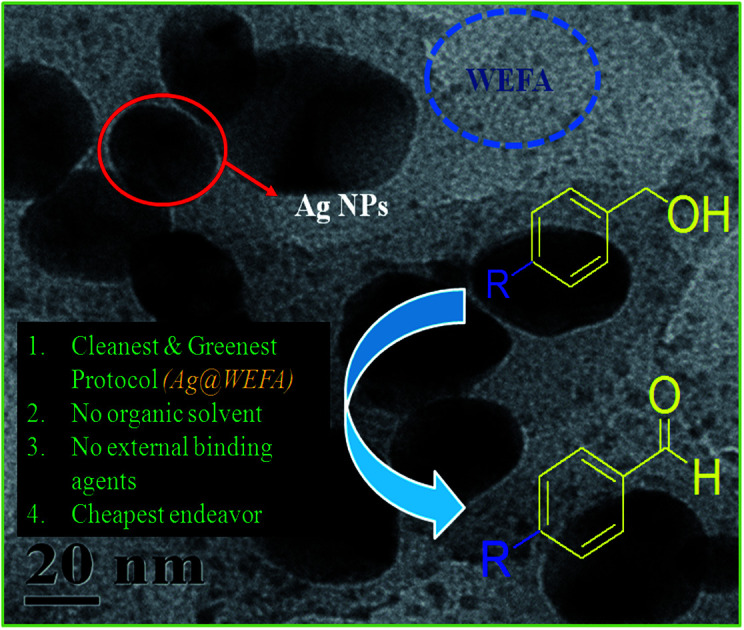

## 1. Introduction

The increasing amount of environmental concerns has led researchers worldwide to develop cleaner and greener reactions for organic transformations. Development of efficient, atom-economical, and selective reactions, which can be performed under safe and mild conditions, has merged out to be one of the prominent subjects in synthetic organic chemistry.

The oxidation of alcohols is one of the fundamental transformations in synthetic organic chemistry.^1,2^ The oxidation products are recognized to be essential intermediates in the manufacture of agrochemicals, fine chemicals, pharmaceuticals, and high-value commodity chemicals.^3^ With the increasing concern for economic and environmental acceptability, researchers across the world are putting significant efforts to accomplish this oxidation reaction with oxygen or hydrogen peroxides.^4,5^ Several excellent catalysts have been developed for environmentally benign oxidation of alcohols to carbonyl compounds. There are reports on the use of Au,^6^ Pd,^7^ Pt,^8^ Co,^9^ Ru,^10^ and Mn^11^ among transition metal catalysts and TEMPO^12^ and mesoporous carbon nitride (mpg-C_3_N_4_)^13^ from the family of metal-free catalysts for these transformations using suitable oxidants such as H_2_O_2_ or molecular oxygen. However, from the safety and environmental point of view, the development of more atom economic catalytic systems that run efficiently without the use of molecular oxygen or hydrogen peroxide would be more attractive. Therefore, oxidative transformation through an oxidant-free dehydrogenative route is of great significance.^14,15^ Dehydrogenative oxidation with the release of hydrogen gas in the absence of any oxidants must be a powerful route from the viewpoint of atom economy. To date, several nanoparticle-based catalytic systems have been reported to exhibit significant applications in these transformations.^16,17^ Hosseini-Sarvari *et al.* prepared nanosized Ag/ZnO catalysts for oxidant-free dehydrogenation of primary and secondary benzyl alcohols to the corresponding aldehydes and ketones under atmospheric pressure.^14^ However, majority of these catalytic protocols have to be carried out under reflux conditions in organic solvents such as toluene. Moreover, they possess some unavoidable limitations such as troublesome catalyst recovery, requirement of acid or base additives, solvents, and involvement of high cost. Importantly, there is a serious need for a greener reaction medium involving lesser impact on environment and having features that are attractive from an industry viewpoint. From green chemistry perspective, it must be very important to develop a new catalytic system that can be used in a greener solvent such as water. In this context, use of water extract of natural feedstock in any organic transformation is of enormous significance as this methodology would eliminate any harsh acids or bases, harmful organic solvents, and other potentially harmful external reagents.^18–21^

Utilization of waste in important organic transformations has been regarded as a significant approach among chemists. Fly ash, a coal combustion residue, has been reported to contain oxides of Si, Al, Ca, Fe, Mg, K, and Na as predominant constituents with trace amounts of B, Mo, Se, Sr, Mn, Ti, *etc.* Numerous organic transformations such as Claisen–Schmidt condensation,^22^ Knoevenagel condensation,^23^ Beckmann rearrangement,^24^ chlorination,^25^*etc.* have been achieved by modification of fly ash as a catalyst. Additionally, silver-based systems have been often used for oxidation of alcohols.^26,27^ Ivanova and co-workers designed Ag/SiO_2_ and utilized it for the oxidant-free dehydrogenation of ethanol.^28^ Moreover, Mitsudome *et al.* successfully fabricated Ag/hydrotalcite catalysts for non-oxidant dehydrogenation of secondary alcohols.^29^ On the basis of these discoveries, we became interested in the design of highly compatible and stable Ag-based catalysts dispersed in waste (water extract of fly ash) and their application in efficient and atom-economical reactions under safe and mild conditions. In the present study, we report an eco-friendly synthetic route to Ag@WEFA for its high catalytic performance in dehydrogenation of alcohols to the corresponding carbonyl compounds. Exhaustive literature survey reveals that NPs dispersed in WEFA have never been reported for any organic transformations. The salient features of the protocol lies in the use of mild environmentally friendly conditions and high yield of the desired products. In addition, the re-use of the catalyst by a very simple method has also been demonstrated.

## 2. Experimental

At first, we planned to design a bio-molecule-assisted synthetic route to Ag NPs taking into consideration their stability and efficiency. Leaves of *Paederia foetida* Linn. were obtained from the National Institute of Technology (NIT) campus, Silchar, Assam, and subjected to washing and drying at ambient temperature for further use.

### 2.1 Materials and physical measurements

Silver nitrate (AgNO_3_) and alcohols were purchased from Sigma Aldrich. Fly ash was obtained from Farakka super thermal power plant situated in West Bengal, India. Double distilled water was used throughout the experiment. FTIR spectra were obtained using a KBr matrix *via* a Bruker 3000 Hyperion Microscope with the Vertex 80 FT-IR system. XRD measurements were carried out using a Bruker AXS D8-Advance powder X-ray diffractometer with Cu-Kα radiation (*λ* = 1.5418 Å) at a scan speed of 2° min^−1^. Transmission electron microscopy images were obtained using a JEOL, JEM 2100 equipment. Scanning electron microscopy images were obtained using a JEOL JSM-7600F FEG-SEM instrument equipped with EDS. ^1^H NMR spectra of the compounds were obtained using a Bruker AV III at 500 MHz.

### 2.2 Preparation of Ag NPs

Ag NPs were prepared with slight modification of our previously reported method.^30^ Leaf extract of *Paederia foetida* Linn. (15 mL) was mixed with 85 mL aqueous solution of (10^−3^ M) AgNO_3_, and the mixture was stirred at room temperature for about 8 h. The mixture changed from initial light yellow to dark brownish; this indicated the formation of colloidal silver. The obtained suspension was centrifuged at 10 000 rpm, followed by oven drying to achieve solid Ag NPs. The reduction of Ag^I^ to Ag^0^ in the process was accomplished by the leaf extract.

### 2.3 Preparation of WEFA (water extract of fly ash)

**Fig. 1:**
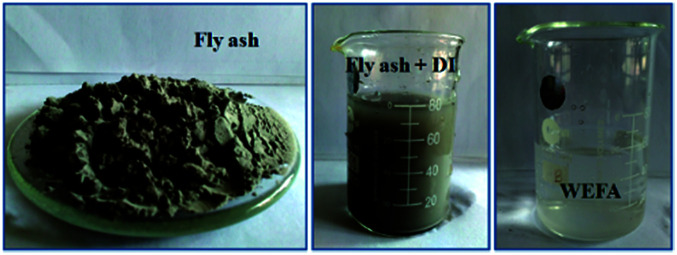
Preparation of WEFA.

WEFA was prepared by adding 50 mL of distilled water to 5 g of fly ash. It was followed by stirring for 10 min and filtration. The filtrate was named as WEFA (Fig. 1).

### 2.4 Preparation of Ag NP-dispersed WEFA (Ag@WEFA)

For Ag@WEFA, 0.5 g of as-prepared Ag NPs was gradually poured into 20 mL WEFA followed by ultrasonication (Selec-TC-533, PCi-Analytics made) for 15 min.

### 2.5 Dehydrogenation of benzyl alcohols using Ag@WEFA

A mixture of benzyl alcohol (1 mmol) and Ag@WEFA (3 mL) was taken and refluxed at 70 °C for an appropriate time. The progress of the reaction was monitored by thin layer chromatography (TLC). After completion of the reaction, the mixture was diluted with 20 mL of water and extracted with 20 mL of diethylether. The combined organic layer was washed with brine and dried over Na_2_SO_4_ and evaporated in a rotary evaporator under reduced pressure. The resulting product was purified by column chromatography to afford the pure product. All compounds are known and have been characterized by comparison of their physical and spectroscopic data with those of authentic samples. A gas burette with a H_2_ sensor was used to confirm the evolution of hydrogen gas from the reaction. Yield percent of the isolated products was calculated using the following equation.Yield% = (actual yield)/(theoretical yield) × 100

## 3. Results and discussions

Previously, Bayat^31^ and his co-researchers had designed magnetically retrievable Ag NPs deposited on the Fe_3_O_4_@SiO_2_ surface and studied their efficacy in oxidant-free dehydrogenation of alcohols. Ag NPs were prepared *via* a chemical route, and tetraethoxysilane (TEOS) was used as a silica source. In addition, stringent and tedious experimental steps were performed for the synthesis of the catalyst. In contrast, our present methodology summarizes the design of a neat catalytic system comprising biosynthesized Ag NPs and water extract of fly ash. The presence of SiO_2_ in high percentages in fly ash also facilitates prevention of agglomeration of Ag NPs and provides a high surface area to stabilize the Ag NPs. Interestingly, Ag@WEFA accelerates the dehydrogenative oxidation reaction to about three folds under mild optimized conditions. It is noteworthy to mention that all the reactions have been efficiently carried out without using any ligand, binding agents (TEOS or CPTES) or hazardous organic solvent.

### 3.1 Characterization of the original fly ash

**Fig. 2:**
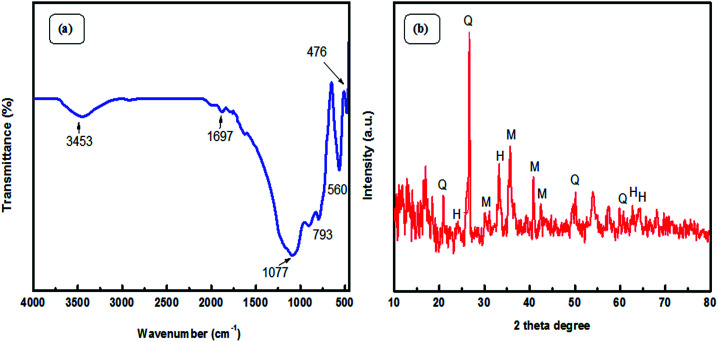
(a) FTIR spectra and (b) XRD patterns of the original fly ash.

**Fig. 3:**
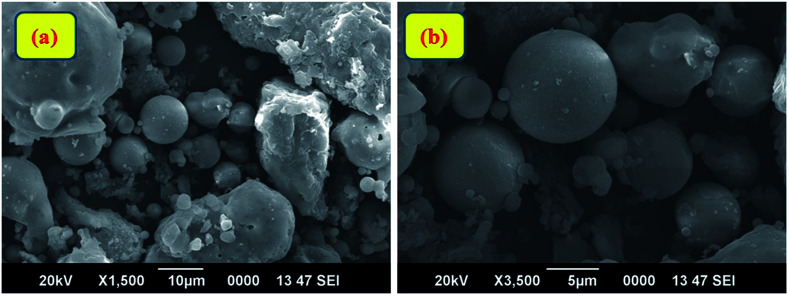
(a) SEM images of the original fly ash and (b) magnified view of the fly ash.

Fourier Transform Infra-Red (FT-IR) spectroscopic analysis was performed to recognize the various functional groups attached to the original fly ash (Fig. 2(a)). The presence of symmetric Si–O–Si stretching and O–Si–O bending vibrations is observed to be at about 793 cm^−1^ and 476 cm^−1^; the absorption bands at 1077 cm^−1^ and 560 cm^−1^ ascertain the presence of asymmetric Si–O–Al and Si–O–Si stretching frequencies, respectively.^32^ Furthermore, a broad absorption peak at 3453 cm^−1^ corresponds to the presence of hydroxyl groups of silanols (–Si–OH).^33,34^ X-ray diffraction (XRD) patterns (Fig. 2(b)) confirmed the presence of quartz, mullite, and hematite phases in the original fly ash.^32^ The peaks are in good agreement with hexagonal quartz (JCPDS card no. 85-1054), orthorhombic mullite (JCPDS card no. 88-0107), and hematite (JCPDS card no. 88-2359). In the SEM images (Fig. 3) of original fly ash, the presence of predominantly spherical particles with smooth surface is evident.

### 3.2 Characterization of the as-prepared Ag NPs

**Fig. 4:**
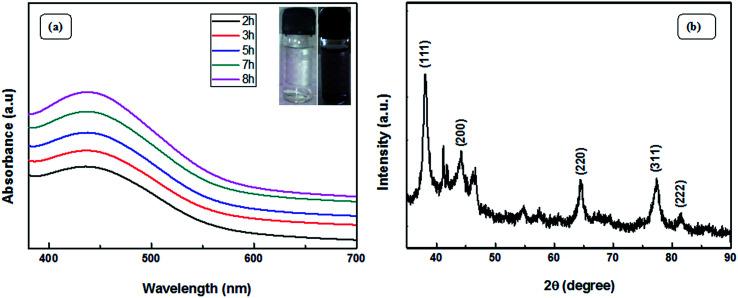
(a) UV-vis spectra of the as-prepared Ag NPs and (b) XRD patterns of Ag NPs.

The visible formation of the Ag NPs was confirmed by ultraviolet-visible (UV-vis) and XRD analysis. Fig. 4(a) shows the UV-vis spectra, which provide evidence for the formation of Ag nanoparticles. Appearance of a distinct peak around 443 nm clearly indicates SPR of Ag nanoparticles. A firm increase in the intensity of SPR with time is observed, which indicates an increase in the yield of the nanoparticles. In the XRD studies, the formation and identification of phases of the synthesized metal nanoparticles were investigated. The XRD patterns of Ag NPs (Fig. 4(b)) shows peaks at 2*θ* equal to 38.19°, 44.27°, 64.79°, 77.70°, and 81.59°, which corresponds to (111), (200), (220), (311), and (222) planes of the face-centered cubic silver system (space group *Fm*3*m*, JCPDS file no. 89-3722).

**Fig. 5:**
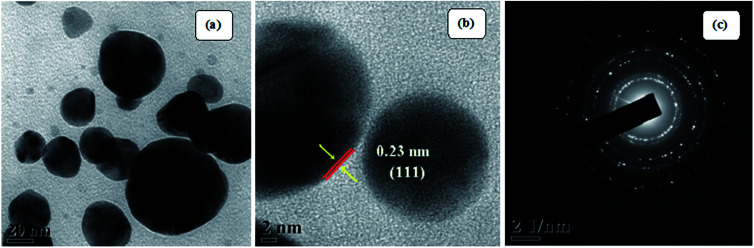
(a) TEM images of the as-prepared Ag NPs, (b) HR-TEM image, and (c) SAED patterns.

Fig. 5 (TEM image) of the as-prepared Ag NPs shows that the particles formed are poly-disperse and consistently spherical with a size range of 10–20 nm. The high resolution transmission electron microscopy (HRTEM) image (Fig. 5(b)) shows that the inter-planar spacing between two adjacent planes is 0.23 nm, which corresponds to the (111) plane of face-centered cubic silver. Selected area electron diffraction (SAED) patterns confirm poly-crystalline nature and shows good concentric rings displaying various planes of face-centered cubic silver (Fig. 5(c)).

### 3.3 Characterization of Ag@WEFA

**Fig. 6:**
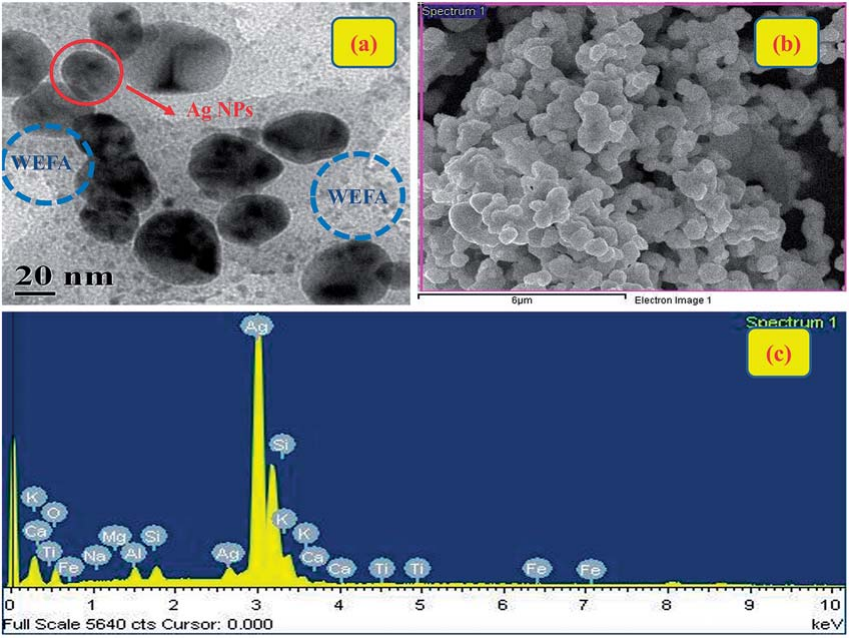
(a) HR-TEM image, (b) SEM image, and (c) EDS spectra of Ag@WEFA.

We have performed HR-TEM, SEM, and energy dispersive X-ray spectroscopy (EDS) analysis to authenticate the formation of Ag@WEFA. The uniform dispersion of 10–20 nm Ag NPs in WEFA was observed in the TEM image (Fig. 6(a)). The dark patches indicate Ag NPs, whereas grey coloured materials in the vicinity of Ag NPs indicate the presence of WEFA. The SEM image (Fig. 6(b)) also shows good dispersion of Ag NPs in WEFA and is consistent with the TEM findings. It must be emphasized that EDS characterization of Ag@WEFA is of critical importance to know the exact composition. Fig. 6(c) shows the presence of Ag, Si, and O in large proportions along with trace amounts of Fe, Ti, Ca, Mg, K, Na, and Al. This is the first ever report on the successful synthesis of NPs dispersed in a water extract of waste-material. It can be claimed to be a very clean and green catalytic system-cum-reaction medium for any transformation.

### 3.4 Catalytic efficiency of Ag@WEFA towards oxidant-free dehydrogenation of benzyl alcohols

The efficiency of the reaction medium (Ag@WEFA) was examined for dehydrogenation of benzyl alcohols to their corresponding carbonyl compounds. To optimize the reaction conditions, 4-methylbenzyl alcohol was taken as the model substrate, and the amount of Ag@WEFA, reaction time, and reaction temperature were studied. From the results of Table 1, we found that 3 mL of Ag@WEFA at a reaction time of 3 h (at 70 °C) procured best results (Table 1, entry 3). The desired product was obtained in very high yield (98%) with high purity. Even 5 mL of Ag@WEFA furnished the reaction in a 96% yield at a reaction time of 2.5 h under identical conditions (Table 1, entry 2). A further increase in the amount of Ag@WEFA to 8 mL with a reaction temperature of 110 °C provided an excellent yield of 97% of the desired product in 1.5 h (Table 1, entry 1). A decrease the amount of Ag@WEFA to 2 mL provided a good 91% yield in 5 h (Table 1, entry 5). At room temperature (RT), a negligible 45% yield was obtained in 7 h (Table 1, entry 6). After exploring a wide range of reaction conditions, it was noted that 3 mL of Ag@WEFA at a reaction temperature of 70 °C and reaction time of 3 h was optimal for carrying out the reactions. It is noteworthy to mention that the amount of Ag dispersed per mL of WEFA is 0.025 g.

**Table 1:** Optimization of the amount of Ag@WEFA, reaction time, and temperature

Sl no.	Ag@WEFA (mL)	Time (h)	Temp (°C)	Yield (%)
1	8	1.5	110	97
2	5	2.5	70	96
**3**	**3**	**3**	**70**	**98**
4	3	2.75	90	95
5	2	5	70	91
6	2	7	RT	45

To explore the scope of this newly designed catalytic system, reactions of a series of benzyl alcohols were conducted under the optimized reaction conditions. Results of the dehydrogenative oxidation of benzyl alcohols in Ag@WEFA are summarized in Table 2. Various substituted benzyl alcohols bearing electron-withdrawing and electron-donating groups showed an excellent reactivity and delivered the desired products in a very good to excellent yield within very short reaction time (Table 2, entries 1–13). Hydrogen gas evolved during the reaction was detected and measured using a gas burette fitted with a H_2_ detector. Conclusively, it was observed that *p*-substituted electron-donating groups give more yield than the electron-withdrawing groups. The ability of this newly designed catalytic system to operate for a wide range of substrates under these low optimized conditions is quite interesting. Therefore, it can be ascertained that our new reaction medium involving Ag@WEFA promises to be an efficient and neat catalytic tool for numerous industrially important organic transformations. The present method offers numerous advantages including high yield, short duration, benign catalytic system *etc.* Furthermore, facile nature of the protocol without use of high temperature, solvent, and special handling procedures is noteworthy. This neat catalytic system thus provides tremendous scope for superior activity with excellent yields as compared to the existing protocols.

**Table 2:** Catalytic dehydrogenation of benzyl alcohols in the presence of Ag@WEFA^a^

Entry	Substrate	Product	Time	Yield (%)
1	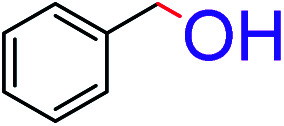	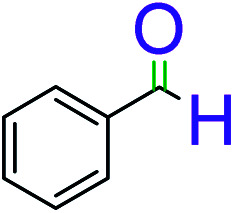	4	96
2	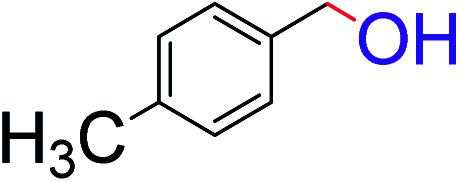	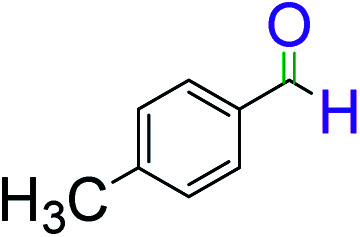	3	98
3	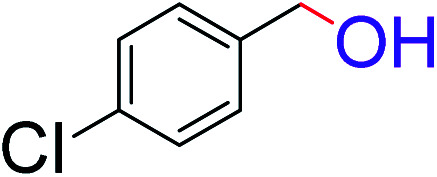	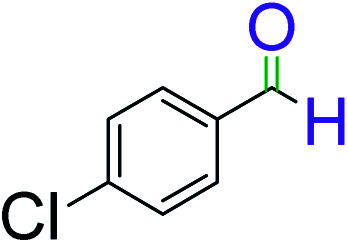	5	94
4	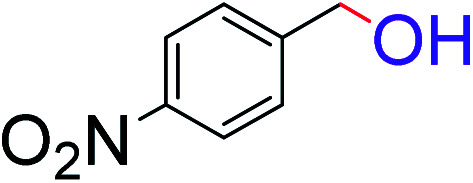	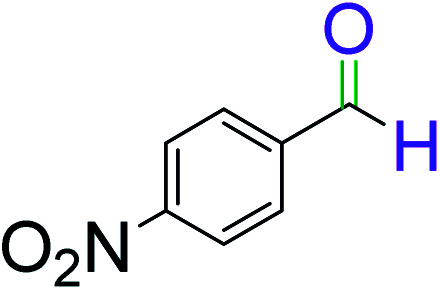	7	93
5	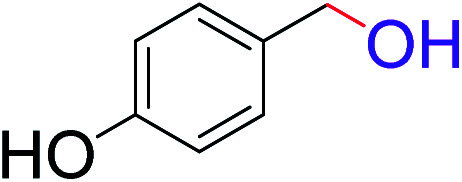	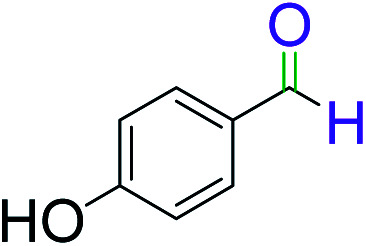	4.5	92
6	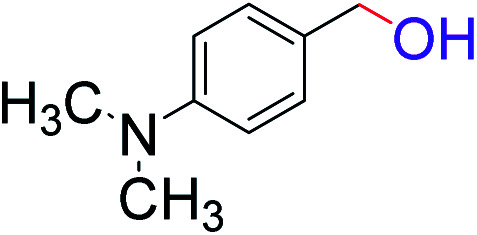	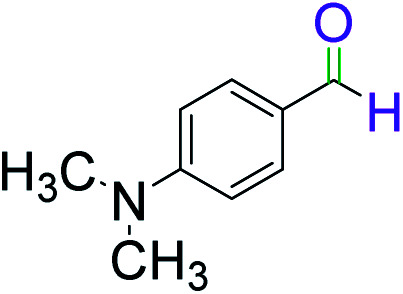	4	97
7	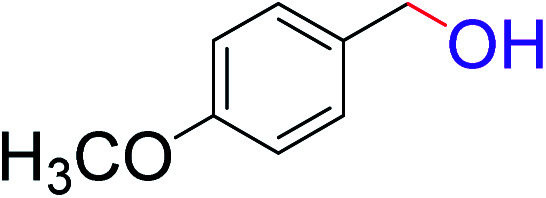	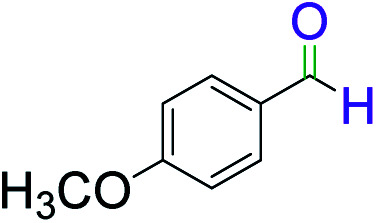	3.5	96
8	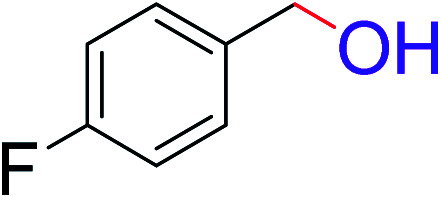	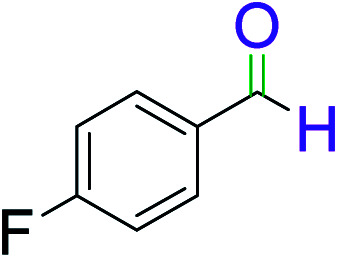	5.5	92
9	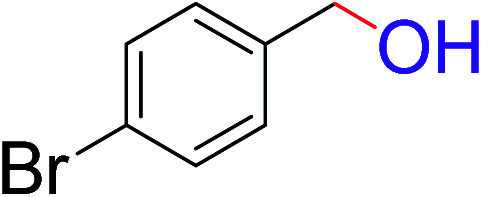	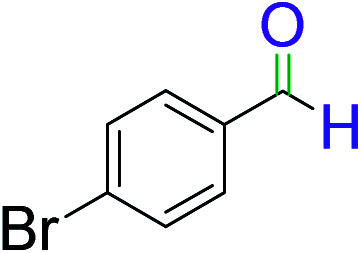	6	90
10	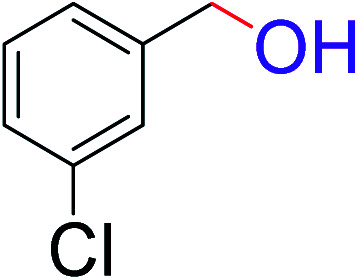	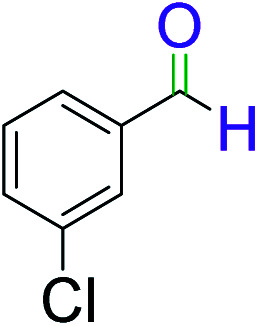	6.5	96
11	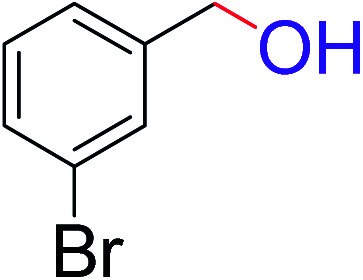	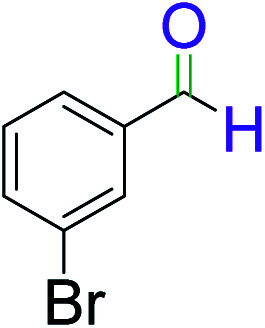	7	93
12	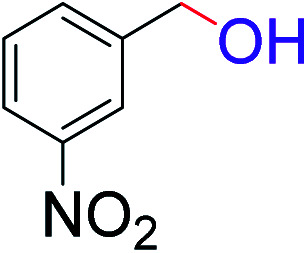	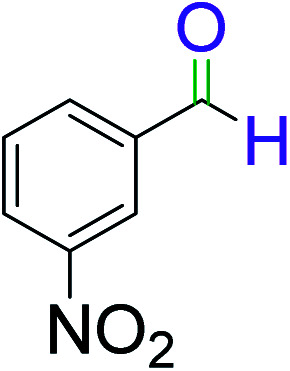	9	92
13	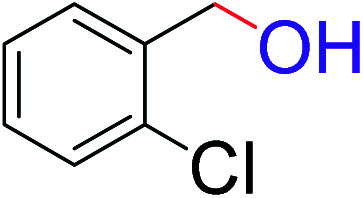	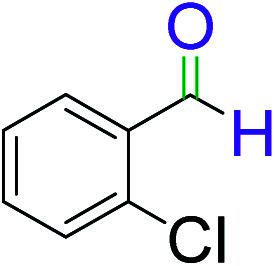	5	95

a Reaction condition: alcohol (1 mmol), Ag@WEFA (3 mL), reaction temperature 70 °C, and N_2_ atmosphere.

**Scheme 1:**
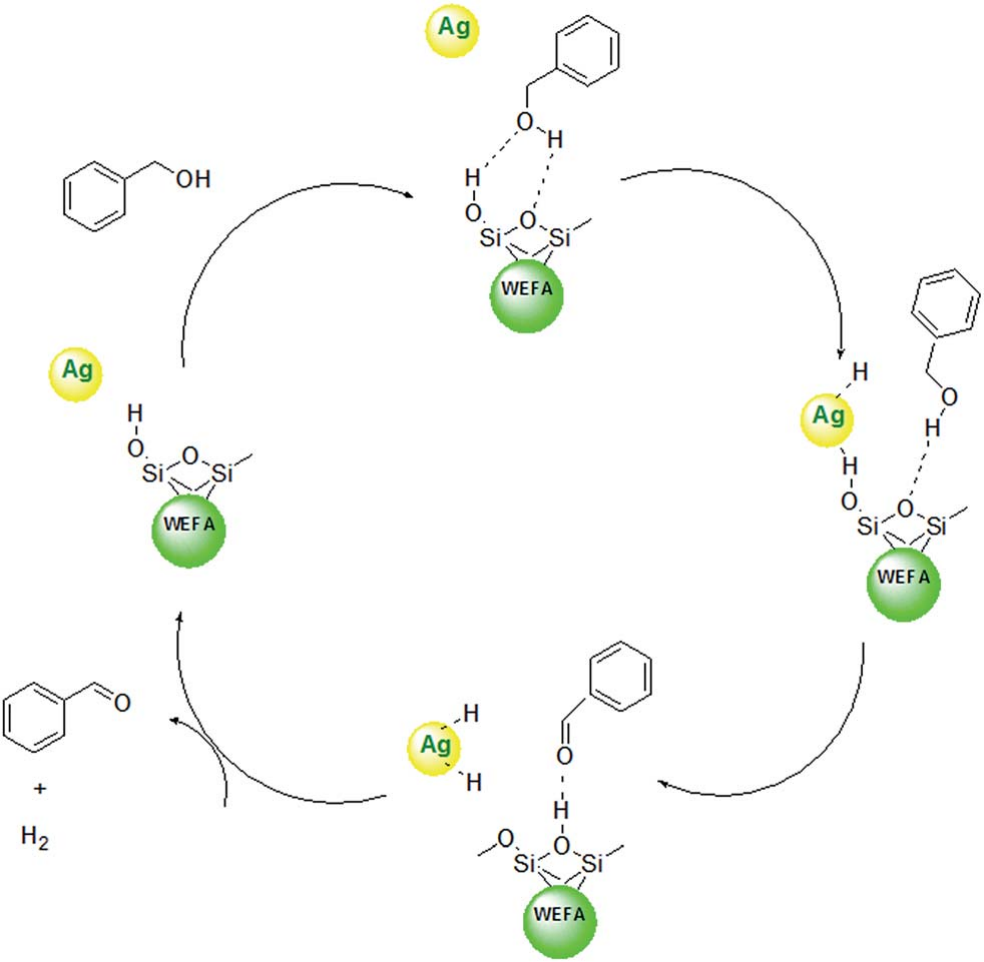
A plausible mechanism for oxidant-free dehydrogenation of benzyl alcohols using Ag@WEFA.

We have definitely observed a dramatic acceleration of the rate of dehydrogenation reaction in Ag@WEFA; however, the mechanism is not fully understandable. However, as previously mentioned, fly ash contains oxides of SiO_2_ along with trace amounts of K_2_O and Na_2_O. Therefore, water extract of fly ash contains Si–OH groups, which interact with benzyl alcohol *via* hydrogen bond interaction. Sequentially, the cleavage of C–H bond takes place on Ag NPs over the surface of WEFA. The proton abstraction from Si–OH groups in WEFA takes place to produce H_2_ adsorbed on the Ag NPs. In the next step, the silica surface of WEFA adsorbs acetaldehyde produced by hydrogen bonds. Finally, H_2_ and benzaldehyde molecules are desorbed from the Ag@WEFA surface; this regenerates the active sites of our Ag@WEFA catalytic system (Scheme 1).^31^

The recyclability of Ag@WEFA was found to be an additional attractive feature of this protocol. We reused Ag@WEFA upto 5th cycle without much significant loss in its efficiency (Table S1 in ESI†). For the recyclability test, 4 methylbenzyl alcohol was taken as a model substrate; after completion of the reaction, products were extracted with diethylether, and Ag@WEFA separated from the product was washed with more diethylether and reused. The retention of the components was evident from the TEM image of the spent catalyst (Fig. S1 in ESI†).

Table 3 provides a comparison between our method and some of the previously reported methods for the dehydrogenation of benzyl alcohols using various catalysts under different reaction conditions. Ru/AlO(OH) and Fe_3_O_4_@SiO_2_–Ag seem to be efficient catalysts as compared to others. However, Ru-based catalysts are known to be costly, and the preparation of Fe_3_O_4_@SiO_2_–Ag undergoes a complex reaction workup, requires use of binding agents, *etc.* Taking into consideration all these factors, our present protocol may be considered as a better, green, efficient, and sustainable catalytic tool for the synthesis of aldehydes *via* oxidant-free dehydrogenation of alcohols.

**Table 3:** Comparison of the literature reported catalysts with present catalyst

Entry	Catalyst	Solvent	Temp (°C)	Time (h)	Yield (%)	Ref.
1	Ru/AlO(OH)	Toluene	80	8	99	35
2	PVP-Ru	Water	Reflux	24	97	36
3	Ag/HT	*p*Xylene	130	10	99	29
4	Ag/Al_2_O_3_	Toluene	100	24	82	26
5	Fe_3_O_4_@SiO_2_–Ag	Toluene	Reflux	24	98	31
6	Ag/ZnO	Toluene	100	8	98	14
7	Ag@WEFA	WEFA	Reflux	3	98	Present work

## 4. Conclusions

In conclusion, a green, efficient, and sustainable protocol for oxidant-free dehydrogenation of benzyl alcohols in Ag@WEFA was developed. The ease of preparation of the catalyst and efficient catalytic transformation in a shortest reaction time without use of any activating agent, ligand or organic solvent definitely adds to the potency of the protocol. To the best of our knowledge, this is the most greener and efficient catalytic tool for oxidant-free dehydrogenation of benzyl alcohols reported to date. Hence, the current study on Ag@WEFA will definitely open new avenues for utilizing these waste materials as neat, robust, and sustainable alternatives for numerous other organic transformations in laboratory as well as in batch scale in near future.

## Conflicts of interest

There are no conflicts of interest to declare.

## Supplementary Material

RA-008-C7RA12225J-s001

## References

[cit1] Choudhary V. R., Dhar A., Jana P., Jha R., Uphade B. S. (2005). Green Chem..

[cit2] Lee A. F., Ellis C. V., Naughton J. N., Newton M. A., Parlett C. M. A., Wilson K. (2011). J. Am. Chem. Soc..

[cit3] Zhou X. T., Ji H. B., Liu S. G. (2013). Tetrahedron Lett..

[cit4] Karimian D., Yadollahi B., Mirkhani V. (2015). Dalton Trans..

[cit5] Davis S. E., Ide M. S., Davis R. J. (2013). Green Chem..

[cit6] Wang J., Lang X., Zhaorigetu B., Jia M., Wang J., Guo X., Zhao J. (2014). ChemCatChem.

[cit7] Nishimura T., Onoue T., Ohe K., Uemura S. (1999). J. Org. Chem..

[cit8] Wang T., Xiao C., Yan L., Xu L., Luo J., Shou H., Kou Y., Liu H. (2007). Chem. Commun..

[cit9] Gawande M. B., Rathi A., Nogueira I. D., Ghumman C. A. A., Bundaleski N., Teodoro O. M. N. D., Branco P. S. (2012). ChemPlusChem.

[cit10] Choi J. H., Kim N., Shin Y. J., Park J. H., Park J. (2004). Tetrahedron Lett..

[cit11] Kwong H. K., Lo P. K., Lau K. C., Lau T. C. (2011). Chem. Commun..

[cit12] Karimi B., Biglari A., Clark J. H., Budarin V. (2007). Angew. Chem., Int. Ed..

[cit13] Su F., Mathew S. C., Lipner G., Fu X., Antonietti M., Blechert S., Wang X. (2010). J. Am. Chem. Soc..

[cit14] Hosseini-Sarvari M., Ataee-Kachouei T., Moeini F. (2015). Mater. Res. Bull..

[cit15] Shimizu K., Kon K., Shimura K., Hakim S. S. M. A. (2013). J. Catal..

[cit16] Marella R. K., Neeli C. K. P., Kamaraju S. R. R., Burri D. R. (2012). Catal. Sci. Technol..

[cit17] Mitsudome T., Mikami Y., Ebata K., Mizugaki T., Jitsukawaa K., Kaneda K. (2008). Chem. Commun..

[cit18] Saikia E., Bora S. J., Chetia B. (2015). RSC Adv..

[cit19] Boruah P. R., Ali A. A., Saikia B., Sarma D. (2015). Green Chem..

[cit20] Boruah P. R., Ali A. A., Chetia M., Saikia B., Sarma D. (2015). Chem. Commun..

[cit21] Saikia B., Borah P. (2015). RSC Adv..

[cit22] Jain D., Khatri C., Rani A. (2011). Fuel.

[cit23] Zhang X., Lai E. S. M., Aranda R. M., Yeung K. L. (2004). Appl. Catal., A.

[cit24] Gopalakrishnan M., Sureshkumar P., Kanagarajan V., Thanusu J., Govindaraju R. (2006). ARKIVOC.

[cit25] Jarmohamed W., Mulder P. (1994). Chemosphere.

[cit26] Shimizu K., Sugino K., Sawabe K., Satsuma A. (2009). Chem.–Eur. J..

[cit27] Geukens I., Vermoortele F., Meledina M., Turner S., Tendeloo G. V., De Vos D. E. (2014). Appl. Catal., A.

[cit28] Sushkevich V. L., Ivanova I. I., Taarning E. (2013). ChemCatChem.

[cit29] Mitsudome T., Mikami Y., Funai H., Mizugaki T., Jitsukawa K., Kaneda K. (2008). Angew. Chem., Int. Ed..

[cit30] Bhuyan B., Paul A., Paul B., Dhar S. S., Dutta P. (2017). J. Photochem. Photobiol., B.

[cit31] Bayat A., Fard M. S., Ehyaei N., Hashemi M. M. (2015). RSC Adv..

[cit32] Mazumder N. A., Rano R., Sarmah G. (2015). J. Ind. Eng. Chem..

[cit33] Tkaczewska E. (2014). J. Ind. Eng. Chem..

[cit34] Zhang Y., Jing L., He X., Li Y., Ma X. (2015). J. Ind. Eng. Chem..

[cit35] Kim W. H., Park I. S., Park J. (2006). Org. Lett..

[cit36] Feng B., Chen C., Yang H., Zhao X., Hua L., Yu Y., Cao T., Shi Y., Hou Z. (2012). Adv. Synth. Catal..

