# Supplementary material for: A silver NP-dispersed water extract of fly ash as a green and efficient medium for oxidant-free dehydrogenation of benzyl alcohols
Source: RSC Adv. 2018 Jan 2;8(3):1313–9. doi: 10.1039/c7ra12225j (PMC9076987; doi:10.1039/c7ra12225j)
Supplement: RA-008-C7RA12225J-s001 [file RA-008-C7RA12225J-s001.pdf]

Electronic Supplementary Material (ESI) for RSC Advances.
This journal is © The Royal Society of Chemistry 2017

Silver NPs dispersed water extract of fly ash as green and efficient medium for oxidant-free dehydrogenation of benzyl alcohols

Bishal Bhuyan, Arijita Paul, Meghali Devi, Siddhartha Sankar Dhar*

Department of Chemistry, National Institute of Technology, Silchar, Silchar-788010, Assam, India

*Corresponding authors. Tel: +91-03842-242915; fax: +91-03842-224797

Email: ssd_iitg@hotmail.com (S.S. Dhar)

Contents **Page Numbers**

TEM image of spent catalyst S1

Spectral & analytical data of compounds S2-S13

Recyclability of the catalytic system comprising Ag@WEFA......................S13

TEM image of spent catalyst:

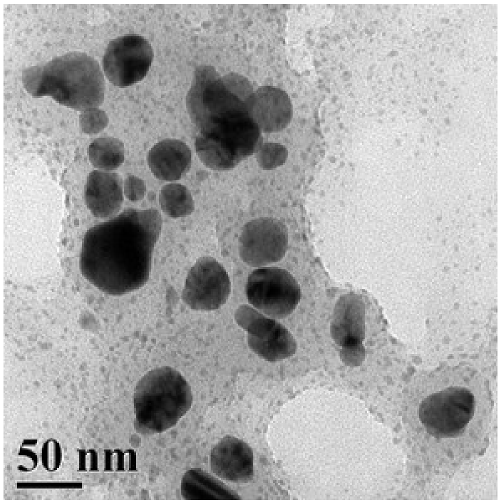

Fig.S1. TEM images of the spent Ag@WEFA catalyst

Spectral & analytical data of compounds:

(1a) Benzaldehyde: Colourless liquid; ^{1}H NMR (400 MHz, $CDCl_3$) δ 9.71 (s, 1H), 7.59 (m, J= 7.4 Hz, 2H), 7.30 (t, 1H), 7.19 (t, 2H).

(2a) 4-Methylbenzaldehyde: Colourless liquid; ^{1}H NMR (400 MHz, $CDCl_3$) δ 9.57 (s, 1H), 7.37 (d, J = 7.2 Hz, 2H), 6.87 (d, J= 7.2 Hz, 2H), 1.96 (s, 3H).

(3a) 4-Chlorobenzaldehyde: Colourless solid; m.p.50°C; ^{1}H NMR (400 MHz, $CDCl_3$) δ 9.98 (s, 1H), 7.83 (d, *J* = 7 Hz, 2H), 7.51 (d, *J* = 6.5 Hz, 2H).

(4a) 4-Nitrobenzaldehyde: Pale yellow solid; m.p.105°C; ^{1}H NMR (400 MHz, $CDCl_3$) δ10.18 (s, 1H), 8.41 (s, 1H), 8.12 (d, *J* = 6.5 Hz, 1H), 8.09 (d, *J* = 6.5 Hz, 1H), 7.30 (t, *J* = 6 Hz, 1H).

(5a) 4-Hydroxybenzaldehyde: Colourless solid; m.p.112°C; ^{1}H (400 MHz, $CDCl_3$): δ =9.83 (s, 1H), 7.80 (d, *J* = 8.4 Hz, 1H), 6.95 (d, *J* = 8.4 Hz, 1H).

(6a) 4-Dimethylaminobenzaldehyde: Yellowish white powder; m.p. 72°C; ^{1}H (400 MHz, $CDCl_3$): δ = 9.79 (s, 1H), 7.79 (d, *J* = 7.5 Hz, 2H), 6.75 (d, *J* = 7.5 Hz, 2H), 3.12 (s, 6H).

(7a) 4-methoxy benzaldehyde: Colourless liquid; ^{1}H (400 MHz, $CDCl_3$): δ 9.86 (s, 1H), 7.83 (d, *J* = 7.0 Hz, 2H), 7.80 (d, *J* = 8.0 Hz, 2H), 3.92 (s, 3H).

(8a) 4-Florobenzaldehyde: Colourless liquid; ^{1}H (400 MHz, $CDCl_3$): δ7.19-7.24 (m, 2H), 7.90-7.94 (m, J = 5.8 Hz, 2H), 9.97 (s, 1H).

(9a) 4-Bromobenzaldehyde: Colourless solid; m.p. 56-57°C; ^{1}H NMR (400 MHz, $CDCl_3$) δ 9.98 (s, 1H), 7.76 (d, *J* = 6.4 Hz, 2H), 7.70 (d, *J* = 6.4 Hz, 2H).

(10a) 3-Chlorobenzaldehyde: Colourless liquid; ^{1}H NMR ($CDCl_3$) δ 9.93 (s, 1H) 7.76 (s, 1H), 7.72 (d, J = 6.5 Hz, 1H), 7.50 (d, J = 7.0 Hz, 1H), 7.42 (d, J = 6.5 Hz, 1H).

(11a) 3-Bromobenzaldehyde: Yellow liquid; ^{1}H NMR (400MHz, $CDCl_3$) δ=9.96 (s, 1H), 7.61 (s, 1H), 7.50 (d, J = 7.5 Hz, 1H), 7.38 (d, J = 7.0 Hz, 1H), 7.11 (d, J = 7.5 Hz, 1H).

(12a) 3-Nitrobenzaldehyde: Yellow solid; m.p. 58°C; ^{1}H NMR ($CDCl_3$) δ 10.13 (s, 1H), 8.71 (s, 1H), 8.49 (d, J = 7.5 Hz, 1H), 8.25 (d, J = 7.5 Hz, 1H), 7.80 (t, J = 7.5 Hz, 1H).

(13a) 2-Chlorobenzaldehyde: Colorless liquid; ^{1}H NMR (400MHz, $CDCl_3$) δ10.33 (s, 1H), 7.78 (m, 1H), 7.75 (m, 1H), 7.34 (m, 1H), 7.27 (m, 1H).

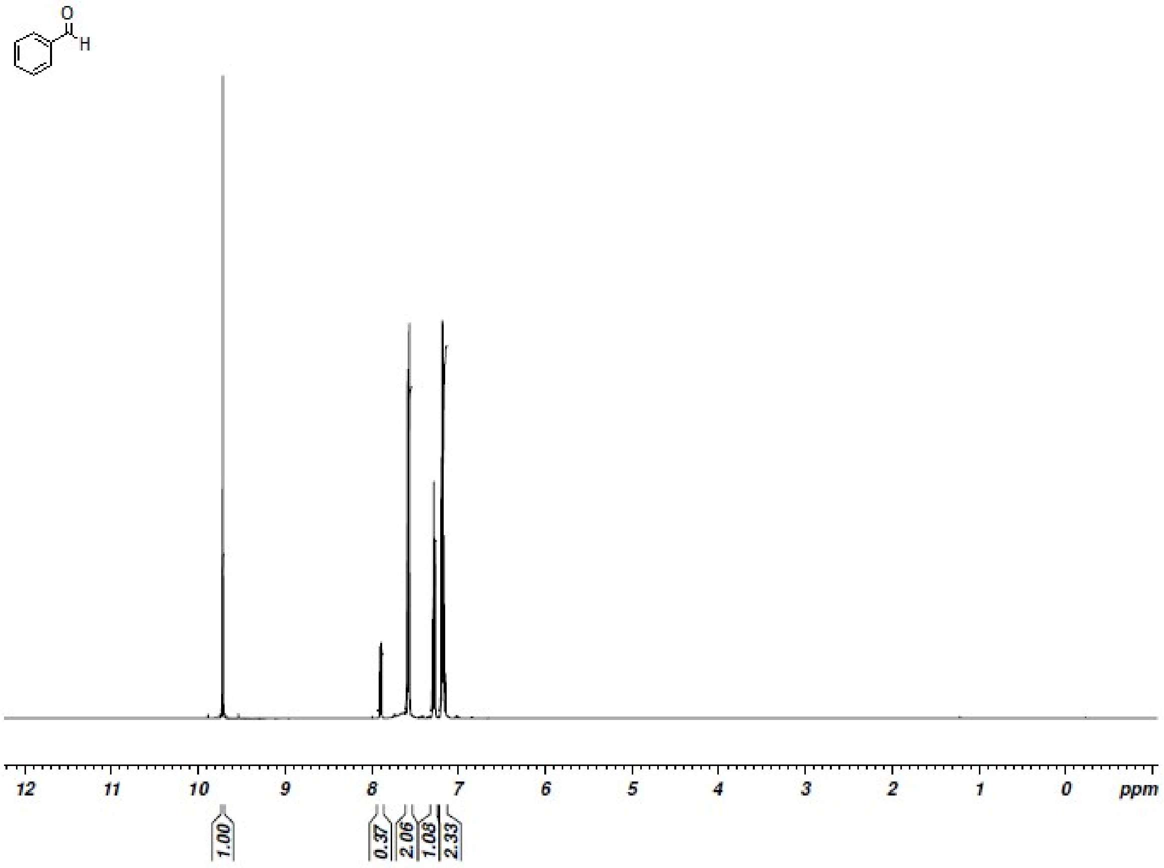

Fig.S2. ^{1}H NMR spectrum of compound (1a) in $CDCl_3$.

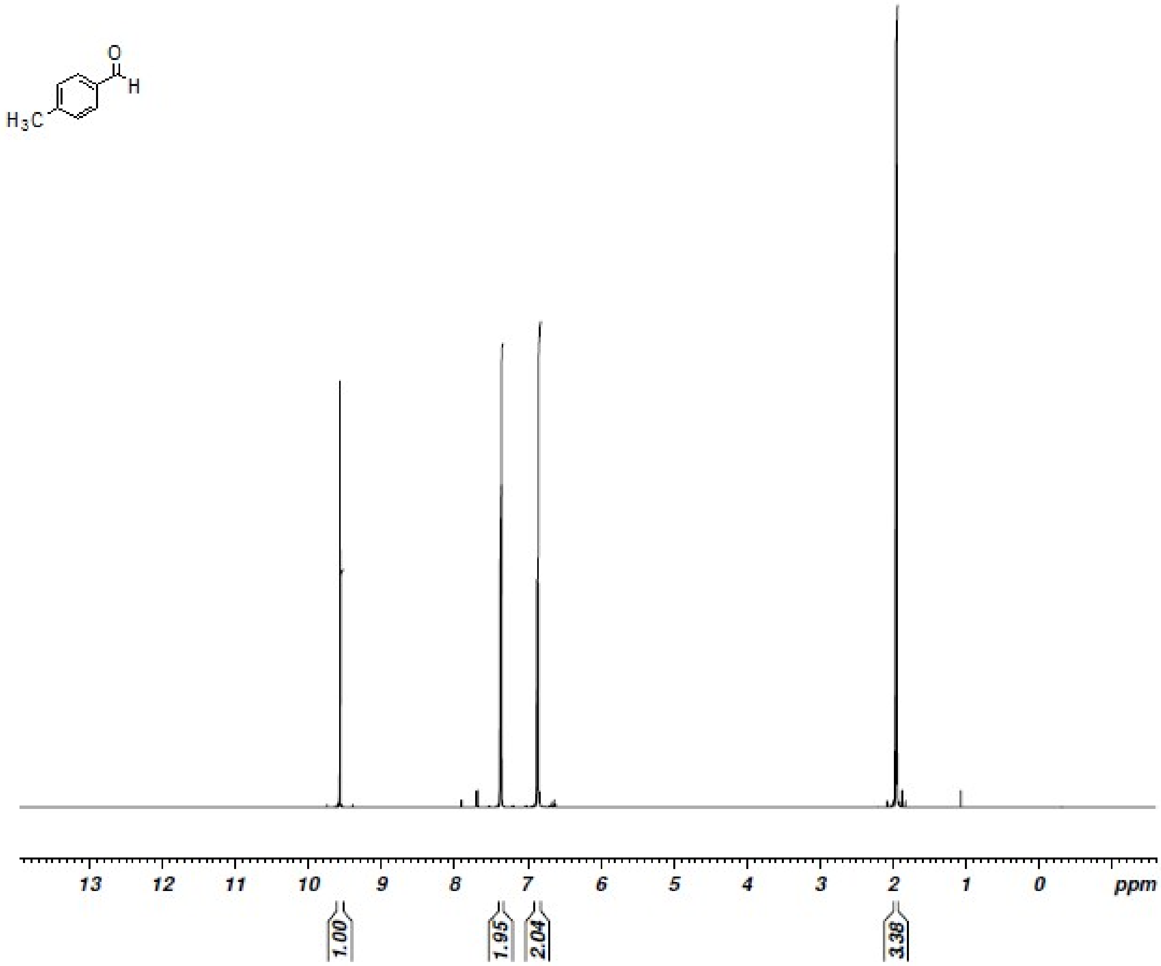

Fig.S3. ^{1}H NMR spectrum of compound (2a) in $CDCl_3$.

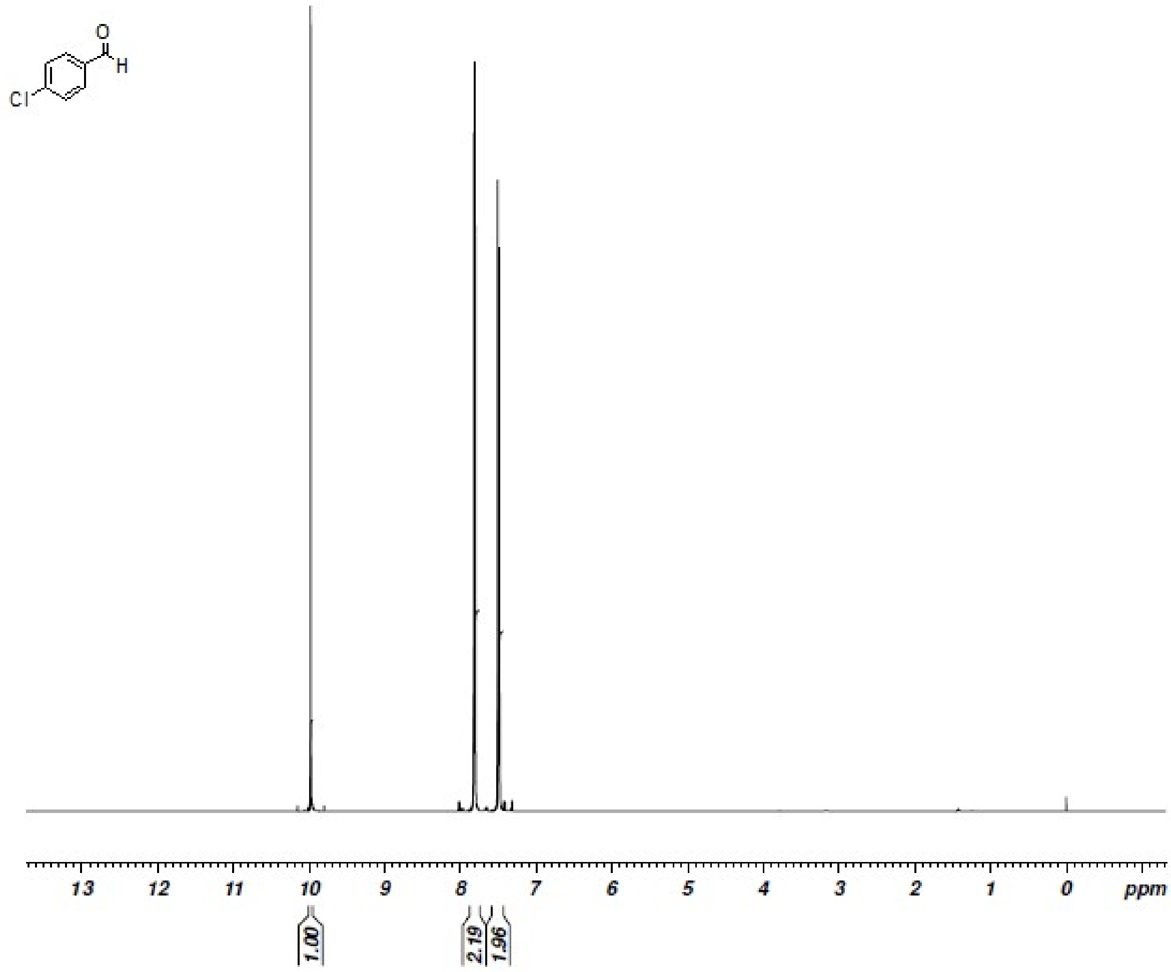

Fig.S4. ^{1}H NMR spectrum of compound (3a) in $CDCl_3$.

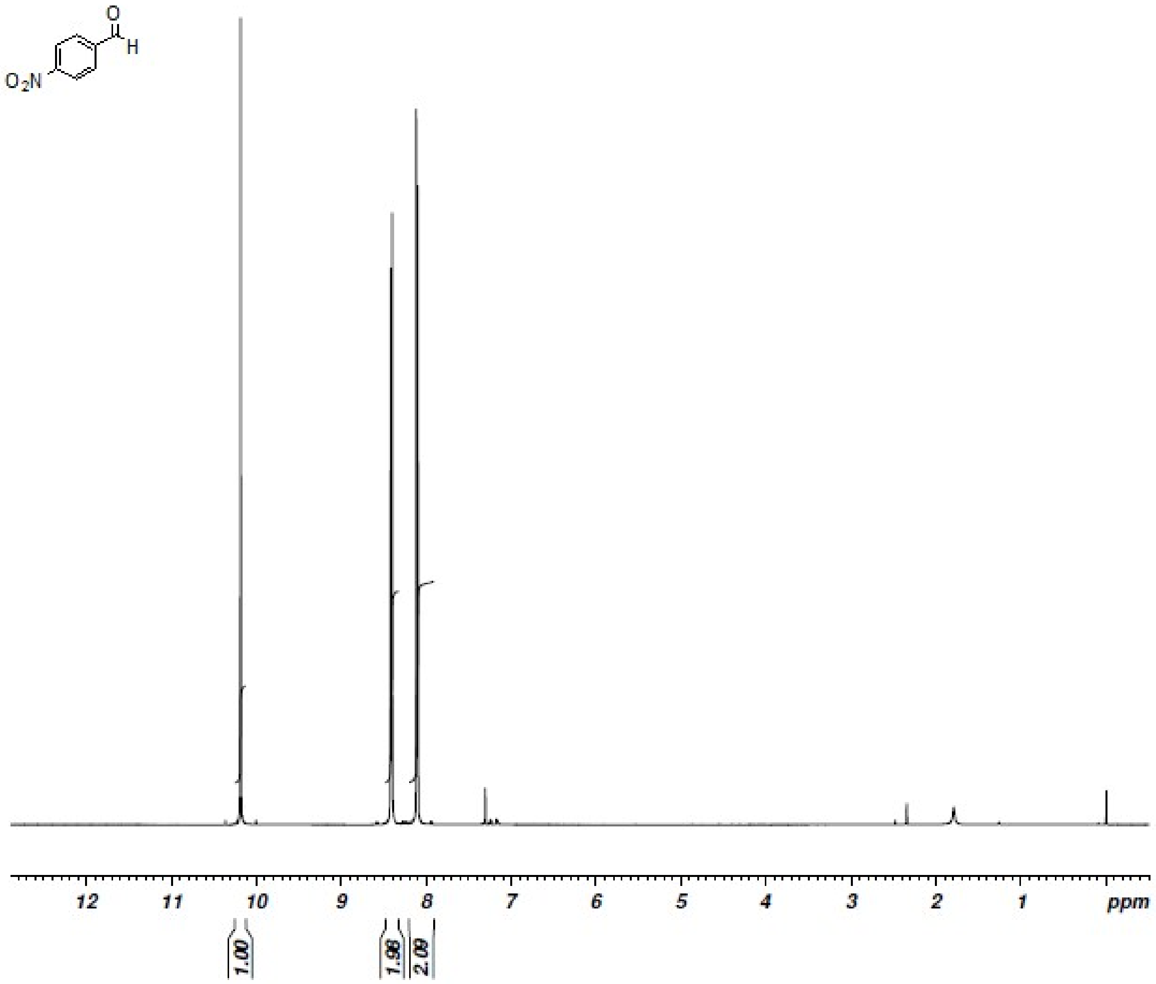

Fig.S5. ^{1}H NMR spectrum of compound (4a) in $CDCl_3$.

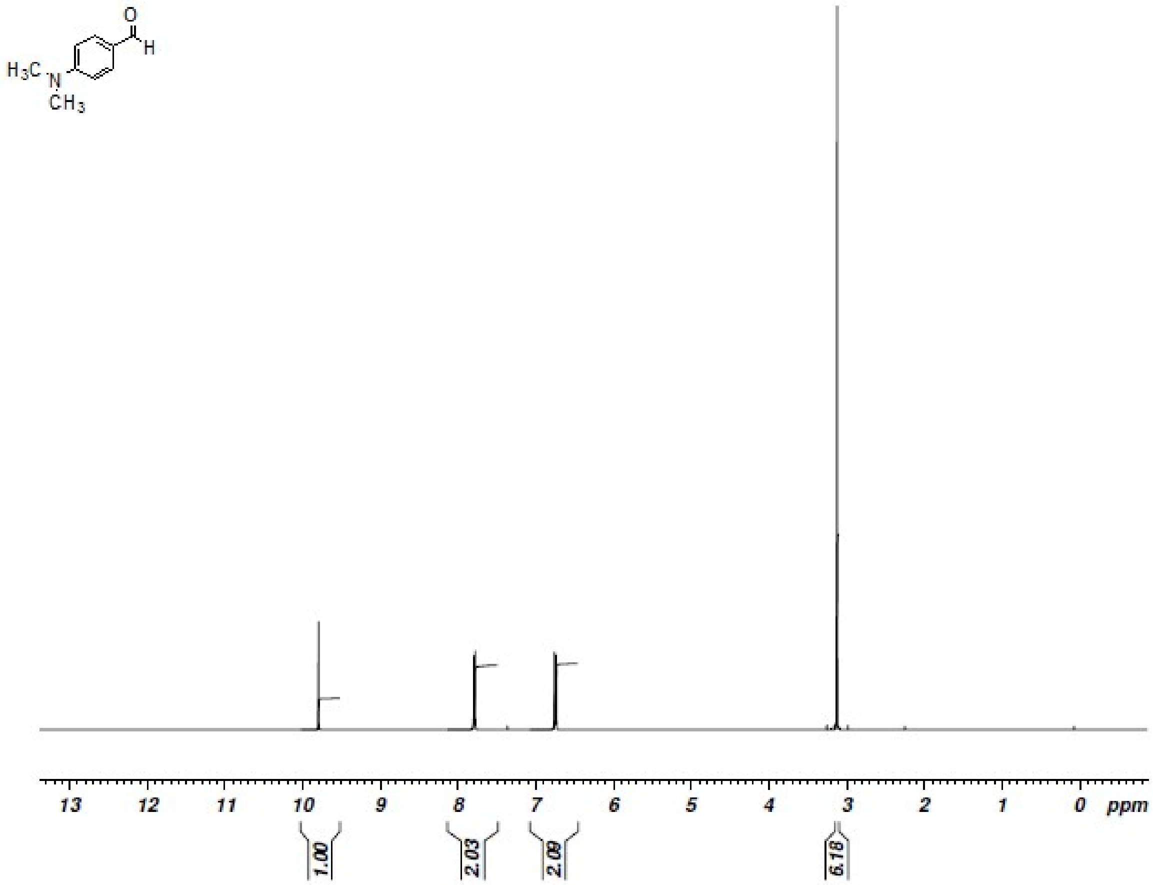

Fig.S6. ^{1}H NMR spectrum of compound (6a) in $CDCl_3$.

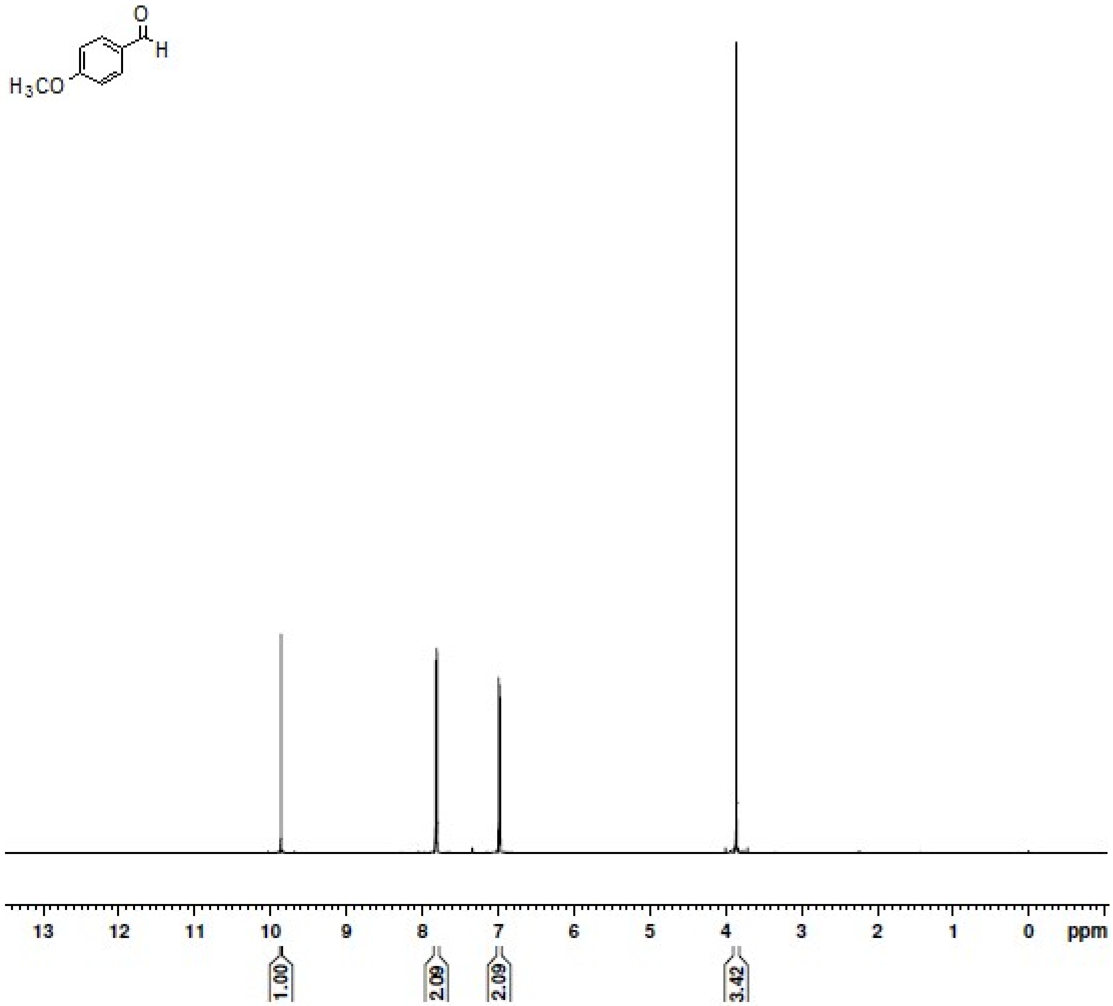

Fig.S7. ^{1}H NMR spectrum of compound (7a) in $CDCl_3$.

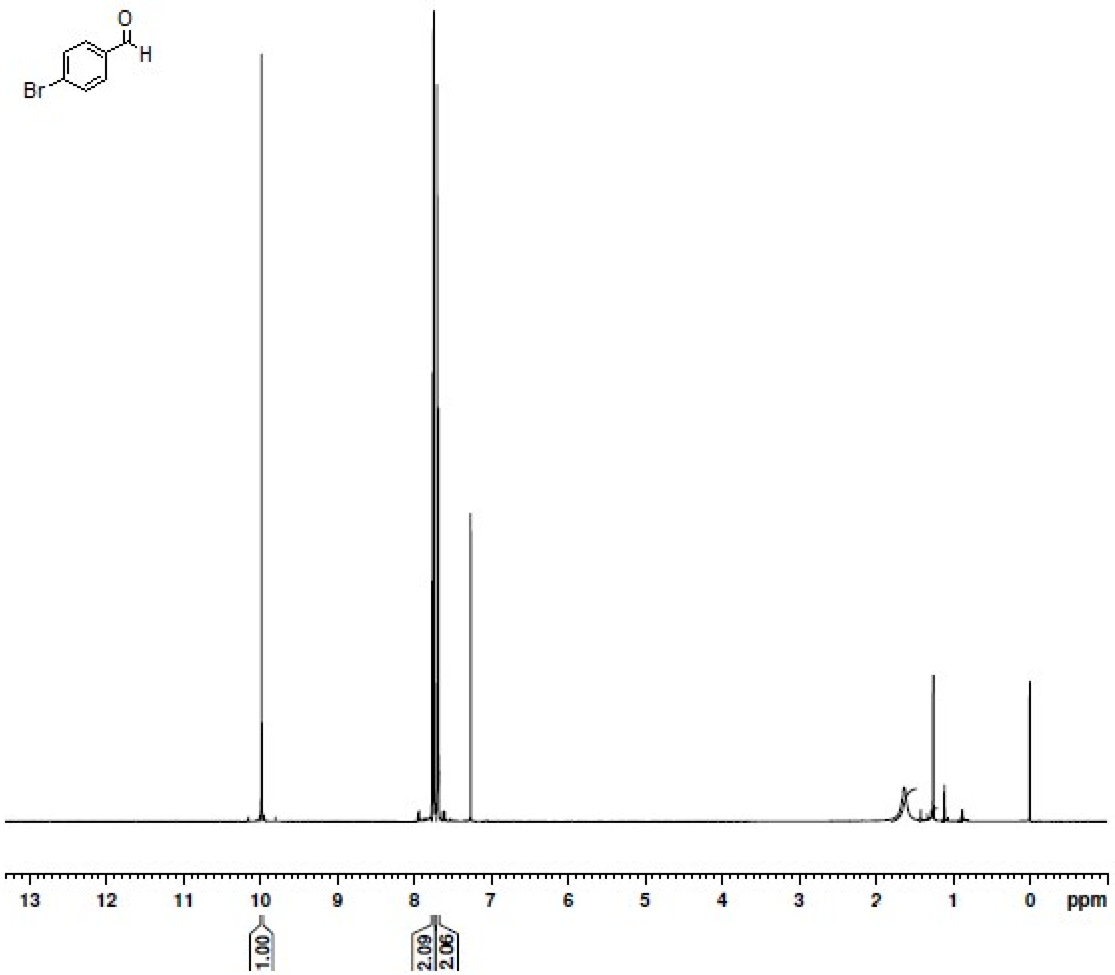

Fig.S8. ^{1}H NMR spectrum of compound (9a) in $CDCl_3$.

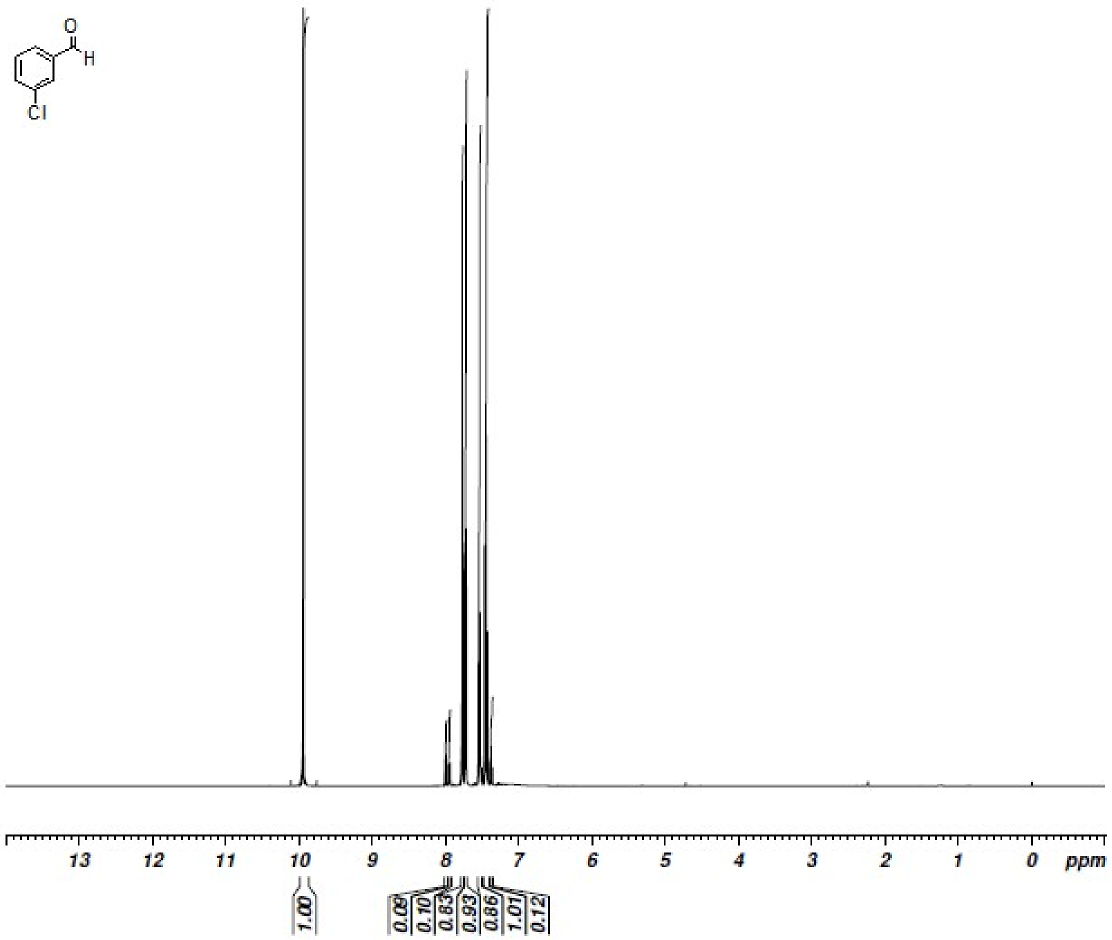

Fig.S9. ^{1}H NMR spectrum of compound (10a) in $CDCl_3$.

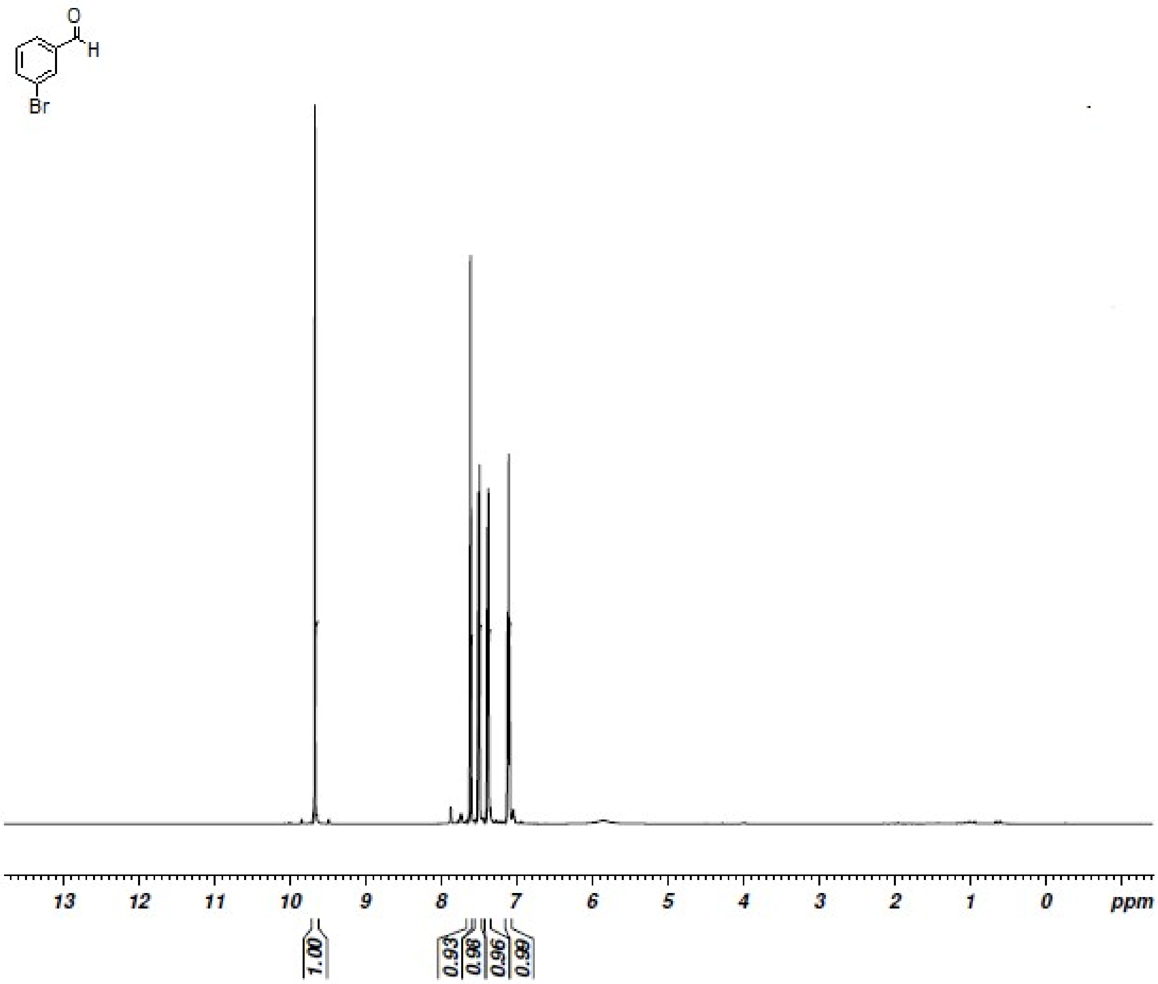

Fig.S10. ^{1}H NMR spectrum of compound (11a) in $CDCl_3$.

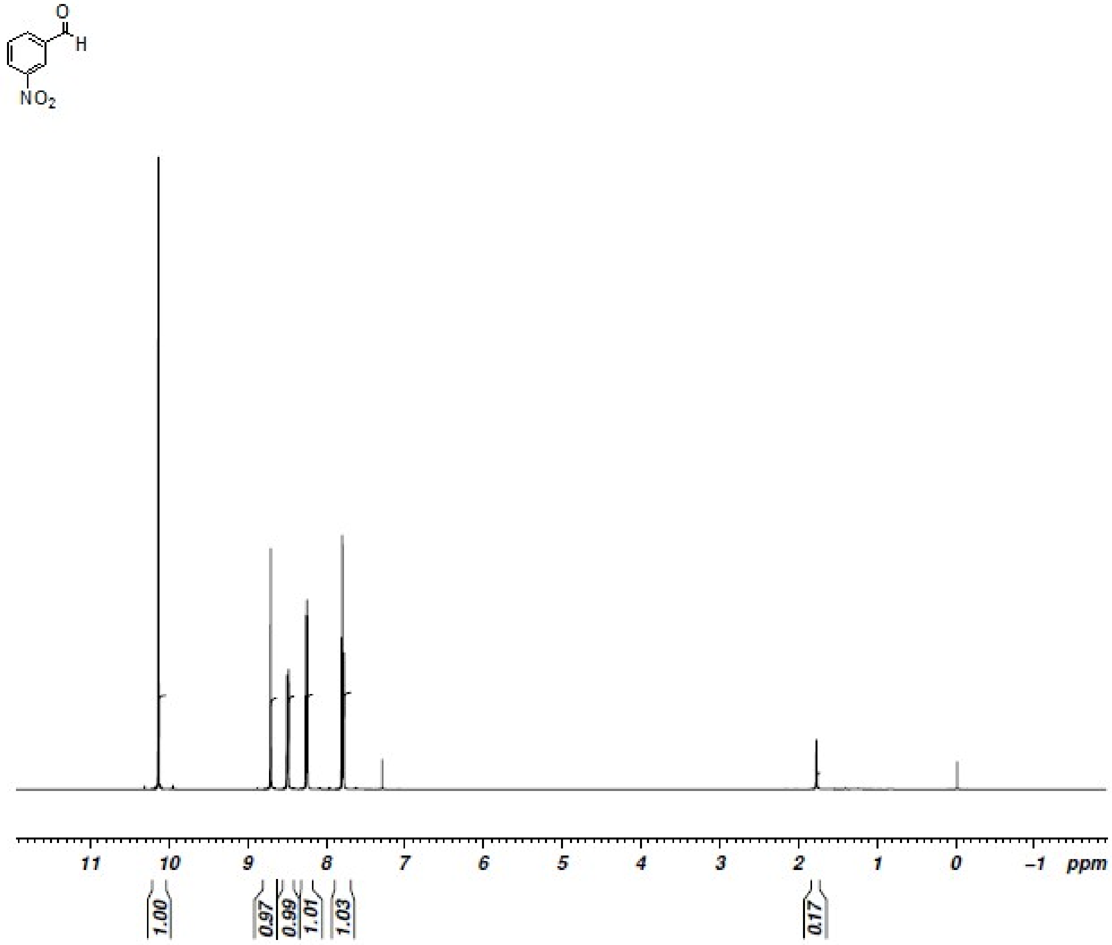

Fig.S11. ^{1}H NMR spectrum of compound (12a) in $CDCl_3$.

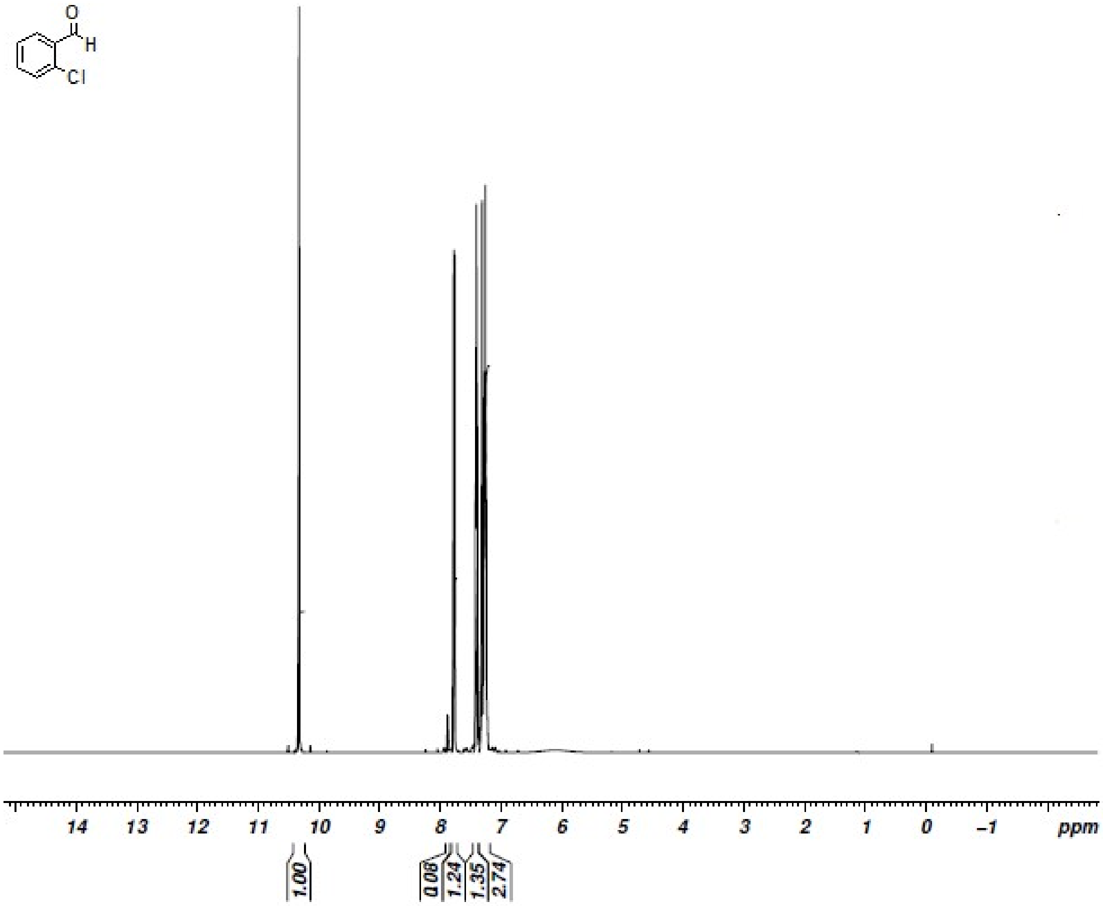

Fig.S12. ^{1}H NMR spectrum of compound (13a) in $CDCl_3$.

Table.S1: Recyclability of the catalytic system comprising Ag@WEFA

Entry	Run	Time (h)	Yield (%)
1	1st	3	96
2	2nd	3	94
3	3rd	3.5	93
4	4th	3.5	92
5	5th	4	90

Reaction conditions: *4-methylbenzyl alcohol (1 mmol), Ag@WEFA (3 mL), reaction temperature 70 °C,* N_2 *atmosphere*
